# Toward Biophysical Mechanisms of Neocortical Computation after 50 Years of Barrel Cortex Research

**DOI:** 10.1093/function/zqaa046

**Published:** 2020-12-21

**Authors:** Carl C H Petersen, Graham W Knott, Anthony Holtmaat, Felix Schürmann

**Affiliations:** 1 Laboratory of Sensory Processing, Brain Mind Institute, Faculty of Life Sciences, Ecole Polytechnique Fédérale de Lausanne (EPFL), Lausanne, Switzerland; 2 Bioelectron Microscopy Core Facility and Brain Mind Institute, Faculty of Life Sciences, Ecole Polytechnique Fédérale de Lausanne (EPFL), Lausanne, Switzerland; 3 Department of Basic Neurosciences and the Center for Neuroscience, University of Geneva, Geneva, Switzerland; 4 Blue Brain Project and Brain Mind Institute, Faculty of Life Sciences, Ecole Polytechnique Fédérale de Lausanne (EPFL), Geneva, Switzerland

Computations in cortical circuits play fundamental roles in higher brain function. Recent technological advances have greatly facilitated the quantitative description of the structure and connectivity of cell-type-specific cortical synaptic circuitry as well as its function in mice carrying out simple goal-directed sensory-perceptual tasks. Mechanistic understanding of how cortical circuits process sensory information requires detailed biophysical computational modeling, in turn demanding increasingly precise data. Through integrative research into structure, function, and simulation, neuroscientists are now in position to investigate causal mechanisms of cortical computation. A key model system for studying neuronal circuit structure–function relationships is the mouse barrel cortex which processes tactile sensory information from the array of whiskers surrounding the snout^1^ (Figure 1A). After 50 years of barrel cortex research since its discovery by Thomas Woolsey and Hendrik van der Loos in 1970,^2^ here, we discuss future research avenues into the Structure, Function, and Simulation of barrel cortex circuits, which will need to be integrated in order to establish causality in structure–function relationships for behavior.

**Figure 1.**
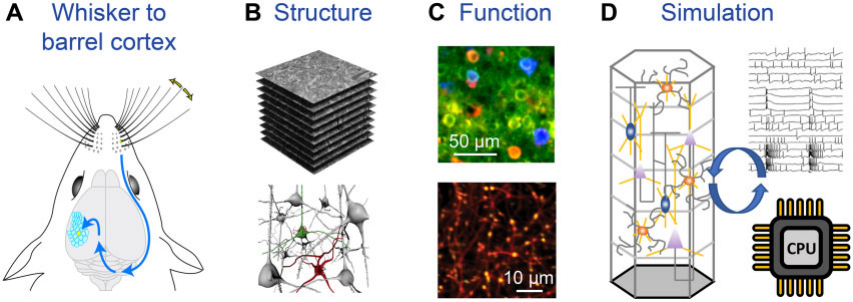
The integration of quantitative structural and functional data relating to barrel cortex circuits in a detailed biophysical simulation is necessary for mechanistic understanding, and generating new hypotheses for further experimental tests. (**A**) Deflection of a facial whisker evokes tactile signals in neurons of the trigeminal ganglion, trigeminal brainstem, somatosensory thalamus, and barrel cortex, where each whisker is individually represented by anatomical units. (**B**) The structure of the barrel cortex can be studied by three-dimensional electron microscopy (above) followed by reconstruction of the neuronal elements (below), which can be identified through correlative light microscopy (unpublished data from Graham Knott). (**C**) In vivo two-photon imaging of calcium-sensitive fluorescent proteins (green) can be used to measure activity in cell bodies (above, red shows retrogradely-labeled barrel cortex cells projecting to secondary somatosensory cortex and blue shows cells projecting to motor cortex, reproduced with permission from Vavladeli et al.^4^) and in axons (below, red shows structure of thalamic axons with yellow indicating high fluorescence from the calcium indicator, unpublished data from Tanika Bawa, Ronan Chéreau, and Anthony Holtmaat). (**D**) Neuronal circuit reconstruction in silico allows simulation of network function through integration of structural and functional data using high-performance computing.

## Barrel Cortex Circuit Structure

The organization of the barrel cortex is such that each column is primarily responsible for processing sensory information from one specific whisker on the snout of the mouse.^1^^,^^2^ Sensory information arrives via the ventral posterior medial thalamus predominantly into layer 4 of barrel cortex, where the barrel-like cell arrangements are apparent. Local microcircuits of various types of excitatory and inhibitory neurons within the barrel cortex process the sensory information in a context- and learning-dependent manner selectively routing signals to downstream brain regions. Long-range synaptic input to barrel cortex from other cortical regions as well as neuromodulatory input, are also likely to play important roles. To understand how the intricate neuronal circuitry of the barrel cortex processes tactile information, it will clearly be important to understand the structure and connectivity of the underlying elements (Figure 1B). A barrel cortex circuit wiring diagram will therefore be essential. Recent advances in imaging and reconstructing all the neuronal wiring of large volumes with electron microscopy^3^ provide hope that in the future it might be possible to establish complete circuit maps of entire cortical columns, or even entire mouse brains. Retaining the molecular identities of cells, axons, and dendrites, however, will also be crucial, and while light microscopy advances toward imaging of entire brains with many labeled cell types, translating these data to the level of the synaptic connections imaged with electron microscopy remains challenging. Combining light and electron microscopy with correlative methods ensures optimum ultrastructural preservation and a multitude of possibilities for specifying the connectivity maps. In addition, the recent refinement of enzymatic electron microscopy labeling methods that can now be genetically-encoded offers new ways to identify the different elements in the barrel cortex. An important future challenge, essential for understanding how barrel cortex processes sensory information, would be to apply these new electron microscopy methods to study thalamic and other long-range synaptic input across layers to the various cell types, as well as defining the local microcircuit connectivity.

## Barrel Cortex Circuit Function

Robust methods for electrophysiological and optical measurement of the activity of projection-specific and genetically-identified cell types in the murine barrel cortex in vivo have begun to provide crucial insight into cortical circuit function during whisker-dependent sensory perception tasks^1^^,^^4^ (Figure 1C). In combination with the latest transgenic and intersectional viral vector technologies, these methods have started to provide insights into the dynamics of neuronal response properties during perceptual learning, for example, showing how specific cell types in barrel cortex respond to various aspects of tactile information during learning of a whisker-based sensory discrimination task.^5^ Many additional questions remain. For example, we still have a poor understanding of the various sensory features that are encoded in the barrel cortex, what are the circuit and cellular mechanisms for synaptic plasticity and dynamic feature encoding during whisker-based learning, and how do long-range neuronal connections, including thalamic input, contribute to learning and perception. Novel optogenetic methodologies that allow the interrogation of specific neuronal circuit components through simultaneous imaging and activation of individual or assemblies of neurons^6^ hold promise to provide insights into the causality of neuronal coding for behavior. Similarly, optogenetic studies combined with single-cell or synapse imaging in vivo will be essential for providing insights into the nonlinear dendritic input–output relationships in barrel cortex neurons,^7^ an important aspect of multisensory integration and long-range input-driven mechanisms for whisker-based perception. Continuous improvements in synaptic mapping and high-resolution in vivo recording techniques will be important for the temporal and spatial assessment of synapse-specific input–output relationships and to test whether the mechanisms for synaptic plasticity that were initially described in brain slices are also at play in barrel cortex circuitry during learning.

## Barrel Cortex Circuit Simulation

Detailed biophysical models of the mechanisms driving cortical function will be an essential component to understanding how cortical circuits work and developing hypotheses for further experimental tests^8^ (Figure 1D). Previous work^9^ demonstrated how neocortical (non-barrel) brain tissue can be reconstructed and simulated in a biophysically detailed computer model despite sparse available data. Recently, Egger et al.^10^ used a biophysical model of the rat barrel cortex to suggest a novel pathway for rapidly processing whisker sensory information in deep cortical layers. Those two studies have in common that they integrated data from rats, where most of the anatomy and electrophysiology was done in the past. In order to benefit from the rapidly increasing experimental data using genetic methods, a natural next step is to extend such modeling approaches to the mouse. Data-driven modeling can help to assign cell identities to the detailed structural data by integrating multiple modalities and constraints, thus providing a quantitative framework to explore how and where the neuronal substrate encodes function. Centering the modeling around genetically-defined cell-types will provide a direct link to the in vivo functional studies as described above, and increased specificity in electron microscopy data will allow further refinement of pathway-specific long-range connectivity, including thalamic input. Model predictions on behavior will require the models to capture brain structures beyond the barrel cortex, but especially for mouse as a model organism, whole-brain biophysical models seem to be in reach in the next years to come.

In summary, the integration of structural and functional data through detailed biophysical simulation promises to provide deep insight into the causal mechanisms of cortical computation. This will necessarily be an iterative process, as increasingly complete data are gathered, allowing increasingly precise simulation giving rise to new hypotheses for experimental testing. Importantly, because the barrel cortex receives input from many brain areas, it may ultimately be necessary to include modeling and measurements across the whole mouse brain in order to obtain a complete understanding. Such large-scale integrative neuroscience research poses a vast challenge for the scientific community, but provides a path for the future development of rational brain therapies for the many brain disorders, which likely, at least in part, result from cell-type-specific circuit deficits.

## Funding

This work was supported by the Swiss National Science Foundation (31003A_182010 to CCHP; 31003A_170082 to GWK; 31003A_173125 to AH; and CRSII3_154453 to AH, GWK and CCHP) and by funding to the EPFL Blue Brain Project (FS) from the Board of the Swiss Federal Institutes of Technology.

## Conflict of Interest Statement

The authors declare no competing interests.
